# The efficacy of acupuncture for generalized anxiety disorder (GAD) in college students: Study protocol for a randomized controlled trial

**DOI:** 10.1371/journal.pone.0316804

**Published:** 2025-01-09

**Authors:** Yang Wang, Xinbo Gu, Baohua Zhi, Yan Yan, Fengyan Lu, Hantong Hu, Quanai Zhang

**Affiliations:** 1 Department of Acupuncture and Moxibustion, The Third Affiliated Hospital of Zhejiang Chinese Medical University, Hangzhou, China; 2 The Third Clinical Medical College, Zhejiang Chinese Medical University, Hangzhou, China; 3 Department of Acupuncture and Moxibustion, The Dingzigu Street Community Health Center, Tianjin, China; Endeavour College of Natural Health, AUSTRALIA

## Abstract

**Introduction:**

Generalized anxiety disorder (GAD) is the most common type of anxiety disorders. GAD usually occurs in adolescence or early adulthood, and the prevalence of GAD is higher among college-enrolled young adults than in the general adult population. However, there is a lack of evidence regarding the effectiveness of acupuncture in the treatment of GAD. This study’s objective is to evaluate the efficacy of acupuncture in the treatment of GAD in college students and its stability.

**Methods:**

A total of 142 subjects will be recruited for the randomized controlled trial (RCT) of current undergraduate and graduate students with GAD and will be randomized into treatment and control groups. The treatment group will receive conventional acupuncture, while the control group will receive sham acupuncture using Streitberger needles. Both groups will be administered 8 acupuncture treatment (2 times per week for 4 weeks). The follow-up duration will be 2 months. The Hamilton Anxiety Scale (HAMA) will be used as the primary outcome of the study to measure the severity of anxiety disorder. The secondary outcomes of this study will be included the Pittsburgh Sleep Quality Index (PSQI) and Self-rating anxiety Scale (SAS). The study will also evaluate the success of blinding and the safety.

**Discussions:**

Findings of this RCT will help evaluate the efficacy of acupuncture treatment for GAD in college students, potentially promoting it as an alternative treatment option. The protocol was registered at clinicaltrials.gov (Identifier: ChiCTR2400080688)

## 1. Introduction

Generalized anxiety disorder (GAD) is the most common type of anxiety disorder, a chronic psychiatric condition characterized by persistent tension and accompanied by autonomic hyperfunction and increased vigilance. GAD usually occurs in adolescence or early adulthood, and the prevalence of GAD is higher among college-enrolled young adults than in the general adult population [1]. WHO World Mental Health Surveys International College Student Project surveyed about 14,000 university students in eight countries and found that the prevalence of GAD was about 18%, second only to major depression [2]. The survey results of 621 university psychological counseling centers in 2018 showed that anxiety was the main reason for students to visit campus psychological health counseling centers [3]. The high prevalence of GAD among college students may reflect the transition from adolescence to adulthood, which is characterized by entering a more independent living environment, increasing personal decision-making responsibilities, and having non family living partners [3]. Anxiety disorders during college have been associated with smoking, sleep problems, and even low academic performance [4]. Meanwhile, there is shown that the presence of anxiety disorders in early adulthood increases the risk of other neurological disorders later in life [5].

The main treatments currently used for GAD include pharmacologic and non-pharmacologic treatments [6]. Pharmacotherapy has some shortcomings while achieving certain therapeutic effects, such as specific adverse reactions, long-term oral easy to lead to drug resistance and addictive, and easy to discontinue the drug withdrawal reaction, which leads to poor adherence to the therapy [7]. Non-pharmacological therapies include psychotherapy, cognitive-behavioral therapy, relaxation therapy, etc., which are affected by many factors and have unstable efficacy, making it difficult for patients to adhere to the treatment for a long time [7]. Therefore, the search for a compliant, safe, and effective treatment is a research trend.

Acupuncture is one of the current therapies for the treatment of GAD [8,9]. As early as the Western *Jin* Dynasty, acupoints for the treatment of mood disorders, such as the *Neiguan* (PC6), were recorded. The "Thirteen Ghost Acupoints" of the *Tang* Dynasty are still highly valued in the treatment of various mental disorders. Clinical trials in recent years have also shown a role for acupuncture in the treatment of GAD [10]. However, there is a study showing that acupuncture has no therapeutic effect on GAD [11]. Therefore, the present study is conducted to evaluate the efficacy and stability of acupuncture in the treatment of GAD in college students with significant health economics.

## 2. Methods

### 2.1 Study design overview

**Fig 1:**
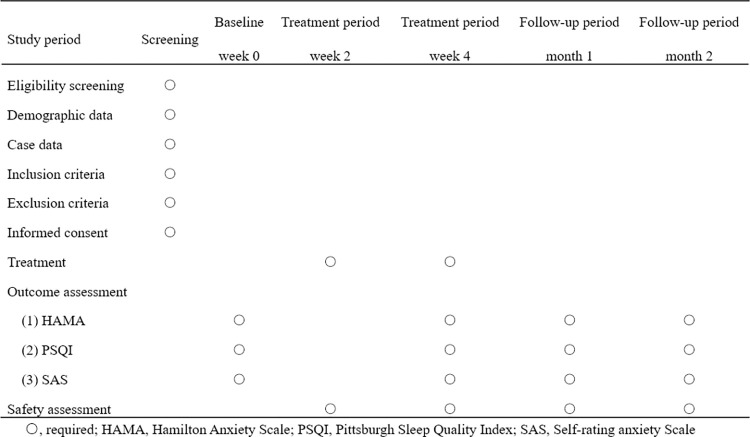
Schedule of enrollment, intervention, and assessment.

**Fig 2:**
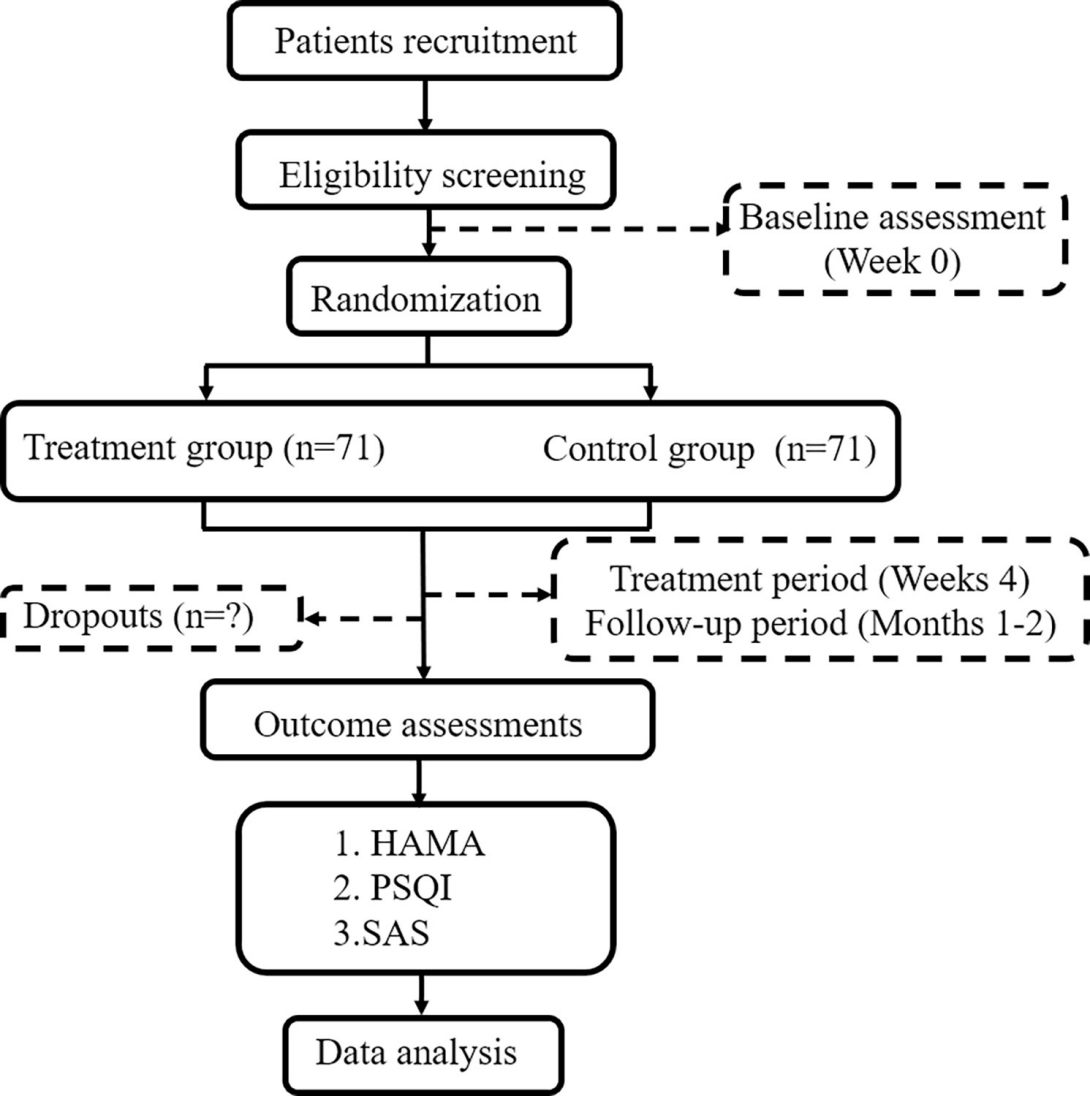
Study flow chart.

This is a single-center, prospective, sham acupuncture controlled, randomized controlled trial. Patients will receive 2 courses of acupuncture + conventional medication or sham acupuncture + conventional medication after randomization. The schedule of enrollment, intervention, and assessment is shown in Fig 1. Each patient will be treated for 4 weeks, with a late follow-up of 2 months; this includes baseline assessment (week 0), end of treatment period II (week 4), end of 1 month of follow-up, and end of 2 months of follow-up (Fig 2). The reporting of this study protocol is based on the Consolidated Standards of Reporting Trials (CONSORT) checklist [12] and the Standards of Reporting Trials and Revised Standards for Reporting Interventions in Clinical Trials of Acupuncture (STRICTA) checklist [13].

### 2.2 Participants: Recruitment and selection

Patients with GAD will be enrolled in the study. The diagnostic criteria based on the Diagnostic and Statistical Manual of Mental Disorders will be used [14]. The following inclusion criteria will be used: current undergraduate and graduate students, those aged 18–30 years; 7≤HAMA total score≤21. Exclusion Criteria: (1) severe organic lesions or current treatments that would make acupuncture unsuitable; (2) anxiety disorders caused by depression or by other psychiatric disorders; (3) inability to cooperate; (4) pregnant and lactating women; (5) participated in other clinical studies in the past 3 months; (6) intolerance to acupuncture treatment.

A total of 142 GAD subjects will be recruited from the acupuncture clinic of the Third Affiliated Hospital of Zhejiang Chinese Medical University through poster advertising. The evaluation of the baseline for the subjects will be conducted within 1 week prior to the first intervention. All subjects will receive written informed consent forms.

All researchers will receive specialized training on the purpose, content, and treatment strategies for quality control. All acupuncturists have at least 5 years of undergraduate education and a certificate of TCM practice. The Monitoring Committee of the Third Affiliated Hospital of Zhejiang Chinese Medical University will monitor the safety of this study and review the results.

This study is approved by the Ethics Committee of the Third Affiliated Hospital of Zhejiang Chinese Medical University (approval number: ZSLL-KY-2023-027-01) and registered at the Chinese Clinical Trial Registry. The relevant information of the study will be provided to all subjects in detail, and they will be given sufficient time to consider whether to participate in the study. If the subjects agree to participate in the study, they will sign an informed consent form. The results of the study will be published at an online medical journal.

### 2.3 Sample size

Following Cohen’s statistical power analysis guidelines, [15] we determine that a medium effect size (d = 0.50) is appropriate for this study, given the absence of preliminary data or prior research that could offer specific effect size estimates. In situations lacking pilot data, selecting a medium effect size is recommended to balance statistical power requirements and resource constraints, while ensuring the ability to detect clinically meaningful differences. Since our primary outcome measure (HAMA score) is a continuous variable that will be compared between groups using means, we applied Cohen’s recommendations for t-tests between independent means. According to these guidelines, 64 subjects per group are required to detect a medium effect size (d = 0.50), with type-I error (α) = 0.05 and power (1-β) = 0.80. Accounting for an anticipated 10% dropout rate, we will recruit 71 subjects per group, resulting in a total sample size of 142 participants. This sample size calculation ensures adequate statistical power to detect clinically meaningful differences between groups while accounting for potential attrition.

### 2.4 Blinding

We will implement blinding for patients, outcome evaluators, and statisticians. The treatment group will be needled at relevant acupoints and *de qi*, while the control group will be needled without breaking the skin and no *de qi*. In the process of treatment, acupuncturists will carry two different types of needles to avoid patients guessing their grouping. In accordance with previous studies using sham acupuncture in this field, [16,17] an independent researcher will evaluate the success of patient blinding using a validated questionnaire. Within 30 minutes after their final treatment, participants will be asked to choose one of three options: "Needled," "Not Needled," or "I Don’t Know". The patients’ responses will indicate whether they are successfully blinded to their treatment allocation. The Bang’s blinding index will be calculated based on the results of this questionnaire, thereby determining the overall effectiveness of our patient blinding protocol.

### 2.5 Randomization and allocation concealment

The randomization will be performed using permuted block randomization with varying block sizes (4, 6, and 8) to ensure balanced group sizes while maintaining allocation unpredictability. The randomization sequence will be generated by an independent statistician using SAS 9.4 software. The generated allocations will be sealed in sequentially numbered, opaque envelopes by an independent research coordinator. Each envelope will contain the group assignment, a unique participant identifier, and date and signature lines for documentation. These envelopes will be stored securely and opened sequentially only after participant eligibility is confirmed and informed consent is obtained. The research coordinator will then open the envelope and inform the acupuncturist of the treatment assignment prior to the participant’s first treatment session, with the date and time of envelope opening being recorded and signed by both the research coordinator and the treating acupuncturist. This process ensures both balanced group sizes and unpredictable allocation while preventing selection bias through proper concealment.

### 2.6 Intervention

(1) The treatment group

During the study period, patients will receive 2 courses of free acupuncture treatment. Treatment will begin on the day patients are randomized into groups. The treatment group will use *Hua Tuo* disposable acupuncture needles (0.25×40 mm, Suzhou Medical Supplies Factory Co., Ltd, Suzhou, China). Patients in the treatment group will choose acupoints including, *Baihui* (GV20), *Yintang* (GV29), *Shenmen* (HT7), *Danzhong* (CV17), PC6, *Taichong* (LR3). The specific anatomical locations of the aforementioned acupoints are detailed in Table 1. GV20, GV29, and CV17 will be punctured 0.3–0.8 inch flat, HT7, PC6, and LR3 will be punctured 0.3–1 inch straight. And leave the needles for 30 minutes after *de qi*. Patients will receive 8 acupuncture treatments (2 times per week for 4 weeks).

**Table 1:** Location of the acupoints.

Acupoints	Anatomical Locations
GV20	On the head, 5 *cun* straight up from the center of the front hairline
GV29	On the head, in the depression between the inner ends of the two eyebrows
CV17	On the chest, transverse to the fourth intercostal space, on the anterior midline
HT7	In the anterior region of the wrist, at the ulnar end of the distal transverse line of the palmar side of the wrist, at the radial margin of the ulnar flexor tendon
PC6	In the anterior region of the forearm, 2 *cun* above the transverse stripe on the distal part of the palmar side of the wrist, between the tendon of the palmaris longus and the tendon of the flexor carpi radialis muscle
LR3	The first and second metatarsal bones on the dorsum of the foot

(2) The control group

Sham acupuncture via Streitberger needles will be used in the control group. With references to similar studies involving sham acupuncture, the selected sham points are the non-acupoints, away from meridians, located on the back, under the spinous processes of the third thoracic vertebra to the seventh thoracic vertebra, and 2 *cun* away from the posterior midline [17]. A total of 8 points on both sides of the back will be subjected to "acupuncture", and the duration of needle retention and the number of treatments will be the same as those in the treatment group. It is worth noting that the use the above non-acupoints in a prone position for the control group is also an attempt to enhance patient blinding.

### 2.7 Outcome measures

#### 2.7.1 Primary outcome

The primary outcome is the evaluation of patient anxiety using the Hamilton Anxiety Scale (HAMA) at weeks 4, at the end of the month 1 of follow-up, and at the end of the months 2 of follow-up. The HAMA total score provides a good response to the severity of anxiety symptoms, and the scale includes 14 items, all of which are rated on a 5-point scale from 0–4. A HAMA *<*7 represents no anxiety; HAMA ≥7 represents possible anxiety; HAMA ≥14 represents definite anxiety; HAMA ≥21 represents significant anxiety; and HAMA ≥29 represents severe anxiety.

#### 2.7.2 Secondary outcomes

a) Pittsburgh Sleep Quality Index (PSQI). PSQI is suitable for evaluating the sleep quality of patients, has 7 main dimensions such as sleep quality, sleep duration, sleep disorders, etc., a total of 18 points self-assessment entries consisted of, and each dimension is scored on a 4-point scale from 0–3. The total PSQI score ranges from 0–21, with higher scores indicating poorer sleep quality. b) Self-rating anxiety Scale (SAS). The SAS is used to assess somatic symptoms associated with anxiety responding. The SAS has 20 items, each of which is rated on a 4-point scale of 1–4. Individual scores on the 20 items are summed, multiplied by 1.25 and rounded to the nearest whole number. A score below 50 is considered normal, 50–59 is considered mild anxiety, 60–69 is considered moderate anxiety, and a score of 70 or more is considered severe anxiety.

### 2.8 Safety assessment

During the trial period, we will systematically collect, assess, report, and manage adverse events (AEs) through both solicited and spontaneous reporting mechanisms. Solicited AEs will be actively monitored using a standardized questionnaire focusing on common acupuncture-associated adverse events (e.g., needling pain, local bleeding, hematoma, dizziness) at each treatment session. Spontaneously reported AEs will be continuously documented throughout the study period based on participants’ voluntary reports. All AEs will be assessed for severity (mild, moderate, or severe), duration, outcome, and their relationship to study intervention (unrelated, possibly related, or definitely related).

The trial clinicians will receive standardized training on AE recognition, assessment, and reporting procedures. Each AE report will be initially evaluated by the treating acupuncturist and subsequently reviewed by an independent safety monitoring committee. For severe AEs, the acupuncturist will provide immediate appropriate care and notify the principal investigator within 2 hours. All severe AEs will be reported to the ethics committee within 24 hours and followed up until resolution. Cumulative AE data will be reviewed monthly by the safety monitoring committee to identify any safety concerns requiring protocol modification. The frequency and severity of AEs will be summarized and reported in the final trial results according to CONSORT guidelines for harm reporting.

### 2.9 Data collection, management, and monitoring

To promote subjects’ retention and complete follow-up, all interventions and outcome evaluation are free to them. Subjects who discontinue interventions but do not drop out will be invited to enter the follow-up period and complete evaluations.

CRF will first be filled out on paper and entered Microsoft Excel by two independent researchers. The reasons for the dropouts and withdrawal of the subjects will be documented throughout the intervention and follow-up period in the CRF. Throughout the entire research process, regular data monitoring and validation will be conducted. The original CRFs and informed consent forms will be kept by the Department of Acupuncture and Moxibustion of the Third Affiliated Hospital of Zhejiang Chinese Medical University, and access will be restricted for 3 years after publication. Without the consent of the principal researcher WY, any original clinical data should not be consulted. The Monitoring Committee of the Third Affiliated Hospital of Zhejiang Chinese Medical University will check the CRF twice a month.

### 2.10 Statistical analyses

The data are analyzed using IBM SPSS (V.25.0), and all statistical tests are taken as two-sided tests. The statistical significance is set at P<0.05. In terms of descriptive statistics, continuous variables will be expressed as mean ± standard deviation, while categorical variables will be expressed as frequencies and percentages.

In terms of comparisons of measurements, as the HAMA, PSQI, and SAS scores are ordinal, non-parametric tests are prioritized for analyzing group differences. For between-group comparisons of HAMA, PSQI, and SAS scores, Mann-Whitney U test will be used; for within-group (pre- and post-test) comparisons, Wilcoxon signed-rank test will be applied. For categorical variables, Chi-square or Fisher’s exact tests will be used as appropriate.

## 3. Discussions

Mental disorders in young people are a worldwide public health problem. Major depressive episodes and GAD are the most common mental disorders among college students [18]. College is the first step toward independent living, and it is a critical period for individuals to form their lifestyles and mental states as they grow up; during this period students must adequately manage their relationships with classmates and roommates outside of their busy academic schedules, and at the same time, Chinese college students face severe employment pressures [19]. Therefore, therapeutic interventions for anxiety sensitivity and psychological distress in GAD patients during this period to develop a healthy psychological state will have a positive impact on their later life [19,20].

There are evidences that acupuncture is effective in the treatment of GAD and relieve patients’ anxiety symptoms [21,22]. Due to the cultural background, it is difficult to blind patients in China. Therefore, we choose the Streitberger needles as the treatment for the control group. The Streitberger needles could simulate the acupuncture process without piercing the skin, [23] and studies have shown its credibility in the effectiveness of acupuncture [24]. Meanwhile, prone position will be used and non-meridian non-points on the back will be selected for further blinding in the control group.

The HAMA is one of the most used rating scales to measure the severity of anxiety symptoms, [25] which is still in use today, and the reliability and validity of the scale are adequate [26]. Therefore, HAMA is the primary outcome of this study. Secondary outcomes are PSQI and SAS. In addition to psychological disorders, patients with GAD exhibit a variety of physical symptoms, such as insomnia, which is characterized by difficulty falling and staying asleep or waking up early; insomnia is associated with the development and progression of GAD [27]. The PSQI is one of the most widely used sleep indicators, and sleep quality scores are correlated with academic performance, mental health, and substance use problems, thus further demonstrating the importance of a good night’s sleep for college students [28]. The SAS that developed in 1971 and still widely used in research, is commonly used to measure physical symptoms associated with anxiety reactions [29]. The SAS has good psychometric properties and is one of the scales used to successfully screen for anxiety disorders [30]. As a self-report questionnaire, the SAS is convenient in assessing anxiety [31].

Major limitations of this study are as follows. First, it is not possible to blind the acupuncturists, which could potentially introduce bias. Second, Streitberger needles are relatively less used in acupuncture clinical trials in China. Although this type of needle is an effective tool to blind participants who receive acupuncture due to its unique design, it may limit the generalizability of our findings to real-world acupuncture practice and research contexts in China.

## 4. Conclusions

In conclusion, this trial will provide a basis for the efficacy of acupuncture in the treatment of GAD in college students as a clinical practice, which will help acupuncturists to make the right decisions when treating GAD in college students.

## Supporting information

S1 Checklist SPIRIT 2013 checklist: Recommended items to address in a clinical trial protocol and related documents*. (DOCX)

S1 File (PDF)

S2 File (PDF)
