# Supplementary material for: The efficacy of acupuncture for generalized anxiety disorder (GAD) in college students: Study protocol for a randomized controlled trial
Source: PLoS One. 2025 Jan 9;20(1):e0316804. doi: 10.1371/journal.pone.0316804 (PMC11717188; doi:10.1371/journal.pone.0316804)
Supplement: S1 File — (PDF) [file pone.0316804.s002.pdf]

针刺治疗大学生广泛性焦虑症疗效影响的随机对照研究

主要叙述国内外同类项目研究现状，市场需求情况和研究意义。可附主要参考文献。

焦虑可以是对压力的一种适当反应，但当它难以控制时则被认为是一种病理性疾病[1]。广泛性焦虑症（Generalized Anxiety Disorder，GAD）是最常见的一种焦虑障碍，以持续性紧张，并可伴有自主神经功能亢进和警惕性增高为特征的慢性精神类疾病。本病是一种常见的致残性疾病，常被低估，同时未被充分治疗；[1] 这会导致沉重的个人痛苦及经济负担[2]。

GAD 通常发生在青春期或成年早期，大学入学的年轻人中 GAD 的患病率高于普通成年人[3]。世卫组织世界心理健康国际大学生项目对 8 个国家约 14000 名大学生进行调查发现 GAD 的患病率约 18%，仅次于重度抑郁症[4]。2018 年对 621 名高校心理咨询中心的调查结果显示，焦虑是学生访问校园心理健康咨询中心的主要原因[5]。大学生 GAD 的高患病率可能反映了青春期向成年期的过渡，其特征是进入更独立的生活环境，增加个人决策的责任，以及拥有非家庭居住伴侣；[5]大学期间的焦虑症与吸烟、睡眠问题甚至学习成绩低下有关[6]。且在早期成年期出现焦虑症，会增加以后患其他神经疾病的风险[7]。因此，预防和治疗大学生 GAD 具有重大的公共卫生意义[8]。

GAD 的发生主要由遗传和环境共同作用，与社会文化、个人体质及心理因素有关[9, 10]。现代医学认为其发病机制与某些神经递质，如 5-羟色胺、γ-氨基丁酸等；[11]大脑前额叶、颞叶功能异常激活；[12, 13]以及神经内分泌功能紊乱有关[14]。

传统医学对郁证的定义是以心情抑郁，情绪不宁，胸部满闷，胁肋胀满，或易怒易哭，或咽中如有异物哽塞等为主症的一类病证。其病因总属情志所伤，发病与肝的关系最为密切，涉及心、脾、肾；基本病机是气机郁滞，脏腑阴阳气血失调[15]。传统医学并无“广泛性焦虑症”一词，但古代文献中记载的“梅核气”“脏躁”“百合病”等都属于本证范畴。早在《黄帝内经》中就有关于情志致病的记载，“人有五藏化五气，以生喜怒悲忧恐。故喜怒伤气，寒暑伤形。暴怒伤阴，暴喜伤阳。”“百病生于气也，怒则气上，喜则气缓，悲则气消，恐则气下，寒则气收，炅则气泄，惊则气乱，劳则气耗，思则气结。”梅核气首见于《南阳

活人书》，《金匮要略》的描述“咽中如有炙脔”。《金匮要略》记录“妇人脏躁，喜悲伤欲哭，象如神灵所作，数欠伸，甘麦大枣汤主之”；“百合病者，百脉一宗，悉致其病也。意欲食，复不能食……如有神灵者，身形如和，其脉微数”。

目前该病主要采用的治疗方法包括药物治疗和非药物治疗；[10]药物疗法在取得一定疗效的同时也存在着诸多缺点，不良反应较多，长期口服易导致耐药性增强并具有成瘾性，同时停药时易出现戒断反应，导致患者对该疗法依从性差；非药物疗法包括心理治疗、认知行为疗法、放松疗法等，非药物治疗受到诸多因素影响而疗效不稳定，病人难以长时间坚持治疗[16]。因此，寻求一种依从性好、安全、有效的治疗方法是研究趋势。

针灸是当前治疗 GAD 的疗法之一[17, 18]。早在西晋皇甫谧的《针灸甲乙经》中就记载了治情志病的穴位有 85 个，例如内关穴主“心澹澹而善惊恐”，然谷穴治疗“心中怵惕，恐如人将捕之状”。唐孙思邈提出“鬼穴十三针”之曲泽、大陵主“心下澹澹喜惊”，少府“主数噫恐悸气不足”；且十三鬼穴至今仍在治疗各种精神类疾病中受到高度重视。近年来临床试验也表明针灸治疗 GAD 有一定作用。姚晓燕等[19]分析了针刺治疗 GAD 的取穴规律，得出结论①强调心脑论治，②主穴可选百会、神门、内关、三阴交、印堂，③循经取穴以督脉、足太阳膀胱经、手厥阴心包经、手少阴心经为主。但也有研究[20]表明针刺对 GAD 无治疗作用。

综上，本课题研究选择具有重要的卫生经济学意义的在校大学生患者作为研究对象，以期评价针刺治疗大学生 GAD 的疗效及其稳定性。

参考文献

[1] DeMartini J, Patel G, and Fancher TL. Generalized Anxiety Disorder[J]. Ann Intern Med, 2019, 170(7):49-64.

[2] Baldwin D, Woods R, Lawson R, et al. Efficacy of drug treatments for generalised anxiety disorder: systematic review and meta-analysis[J]. BMJ, 2011, 342:1199.

[3] Lijster JM, Dierckx B, Utens EM, et al. The Age of Onset of Anxiety Disorders[J]. Can J Psychiatry, 2017, 62(4):237-246.

[4] Auerbach RP, Mortier P, and Bruffaerts R, et al. WHO World Mental Health

Surveys International College Student Project: Prevalence and distribution of mental disorders[J]. J Abnorm Psychol, 2018, 127(7):623-638.

[5] Byrd-Bredbenner B, Eck K, and Quick V. Psychometric Properties of the Generalized Anxiety Disorder-7 and Generalized Anxiety Disorder-Mini in United States University Students[J]. Front Psychol, 2020, 24(11):550533.

[6] Bartolo A, Monteiro S, and Pereira A. Factor structure and construct validity of the Generalized Anxiety Disorder 7-item (GAD-7) among Portuguese college students[J]. Cad Saude Publica, 2017, 33(9):e00212716.

[7] Strohle A, Gensichen J, and Domschke K. The Diagnosis and Treatment of Anxiety Disorders[J]. Dtsch Arztebl Int, 2018, 155(37):611-620.

[8] Kanuri N, Taylor CB, Cohen JM, et al. Classification models for subthreshold generalized anxiety disorder in a college population: Implications for prevention[J]. J Anxiety Disord, 2015, 34:43-52.

[9] 张心保.神经精神病学［M］.北京：科学技术出版社,1980：87.

[10] Hoge EA, Ivkovic A, and Fricchione GL. Generalized anxiety disorder: diagnosis and treatment[J]. BMJ, 2012, 345: e7500.

[11] Marcinkiewcz CA, Mazzone CM, D’Agostino G, et al. Serotonin engages an anxiety and fear-promoting circuit in the extended amygdala[J]. – Nature, 2016, 537(7618):97-101.

[12] Ball TM, Ramsawh HJ, Campblee-Sills L, et al. Prefrontal dysfunction during emotion regulation in generalized anxiety and panic[J]. Psychol Med, 2013, 43(7):1475-86.

[13] Robinson OJ, Krimsky M, Lieberman L, et al. Towards a mechanistic understanding of pathological anxiety: the dorsal medial[J]. Lancet Psychiatry, 2014, 1(4):294-302.

[14] Hibert K, Lueken U, and Beesdo-Baum K.H. Neural structures, functioning and connectivity in Generalized Anxiety Disorder and interaction with nerurendocrine systems: a systematic review[J]. J Affect Disord, 2014, 158:114-26.

[15] 吴勉华, 王新月. 中医内科学[M]. 3 版. 北京：中国中医药出版社, 2014:351-358.

[16] 赵晶, 全亚萍, 陈园桃. 广泛性焦虑症中医药研究进展[J]. 辽宁中医药大学学报, 2015, 17(04): 210-212.
[17] 于学平, 张庚鑫. 水沟穴治疗广泛性焦虑症疗效观察[J]. 上海针灸杂志, 2016, 35(02):162-164.
[18] 赵岩, 邹伟, 滕伟, 等. 通督调神针刺法治疗广泛性焦虑症的临床研究[J]. 针灸临床杂志, 2014, 30(11): 24-26.
[19] 姚晓燕, 刘军, 李珊珊, 等. 针刺治疗广泛性焦虑症取穴规律研究[J]. 中国中医药信息杂志, 2017, 24(07): 94-97.
[20] Mark AD, Chung VCH, Yuen SY, et al. Noneffectiveness of electroacupuncture for comorbid generalized anxiety disorder and irritable bowel syndrome[J]. J Gastroenterol Hepatol, 2019, 34(10):1736-1742.

研究内容

主要叙述课题研究内容、拟解决关键问题、预期目标及主要创新点等。

1 研究内容

本研究以大学生 GAD 为研究对象，拟收集符合纳入标准的患者，采用随机对照试验（RCT）的方法，将患者随机分入 2 组接受治疗，分别为针刺组及对照组，以汉密尔顿焦虑量表(HAMA) 、匹兹堡睡眠质量指数（PSQI）及焦虑自评量表（SAS）对疗效进行评价，探索针刺治疗 GAD 的疗效。

2 拟解决的关键问题

在治疗大学生 GAD 的方法中，针刺是否为一种有效方法。

3 预期目标

明确针刺是否能够成为治疗大学生 GAD 的有效方法，及其疗效的稳定性。

4 主要创新点

本研究观察针刺治疗前后大学生 GAD 患者各项症状量表的变化，为针刺是否能够改善大学生 GAD 症状提供一定的依据。

研究方法和技术路线

主要叙述课题的研究方法、技术路线和可行性分析等。

1 研究方法

1.1 研究对象

1.1.1 受试者来源

本课题的研究对象均来自于我院针灸科且符合意向各项标准的患者。

1.1.2 诊断标准

（1）西医诊断标准参照《精神障碍诊断与统计手册（DSM-5）》（精神障碍诊断与统计手册 第 5 版[S].北京:北京大学出版社, 2015.7）中 GAD 的诊断标准：

① 个体难以控制这种担心；

② 这种焦虑和担心与下列 6 种症状至少 3 种有关：1.坐立不安或感到激动或紧张。2.容易疲倦。3.注意力难以集中或头脑一片空白。4.易激惹。5.肌肉紧张。6.睡眠障碍。

③ 这种焦虑、担心或躯体症状引起有临床意义的痛苦，或导致社交、职业或其他重要功能方面的损害。

④ 这种障碍不能归因于某种物质的生理效应，或其他躯体疾病。

⑤ 这种障碍不能用其他精神障碍来更好地解释。

⑥ 在至少 6 个月的多数日子里，对于诸多事件或活动，表现出过分的焦虑和担心。

（2）中医诊断标准参照全国高等中医药院校规划教材《中医内科学》郁病诊断标准：

① 以忧郁不畅，情绪不宁，胸胁胀满疼痛为主要症状，或有易怒易哭，或有咽部异物感，吐之不出，咽之不下的表现；

② 有情绪创伤，焦虑，悲伤，恐惧，怨恨和其他情绪内伤的病史，并且反复发作的疾病通常与情绪因素密切相关；

③ 无其他症状和体征；

④ 符合以上标准并持续 6 个月以上者。

1.1.3 纳入标准

（1）与 GAD（郁病）中西医诊断标准相符者；

（2）生命体征平稳，意识清醒，无自杀倾向者；

（3）在读本科、研究生，18≤年龄≤30 岁者；

（4）7≤HAMA 总分≤21 分；

（5）自愿接受治疗及观察，并签署知情同意书者。

1.1.4 排除标准

（1）严重器质性病变或存在不适合当前治疗方法者；

（2）抑郁症或其他精神类疾病继发焦虑症者；

（3）神志异常不能合作者；

（4）孕妇及哺乳期妇女；

（5）近 3 个月参加过其它临床研究者；

（6）不能耐受针灸治疗者。

1.1.5 剔除标准

（1）受试者依从性差，纳入后未配合试验方案完成治疗者；

（2）观察中自行脱落病例或自行退出研究者。

1.1.6 脱落标准

（1）在治疗过程中出现晕血、晕针、断针、滞针等要求停止治疗未能完成疗程；

（2）失访。

1.1.7 病例中止标准

（1）在治疗过程中出现严重并发症、严重不良反应不宜继续试验者；

（2）中途因为其他外界原因主动提出退出本临床研究的受试者。

2 研究方案

2.1 样本量估算

按照临床试验最小样本量原则选取每组30例受试者，并按照小于20%的失访率，每组纳入受试者36例，研究样本总量为72例。

2.2 随机方法

研究采用完全随机、对照、单盲法进行试验，将 72 名受试者随机分为两组（每位受试者根据前来就诊的先后顺序进行 001-072 的序号编号，然后用 SPSS 25.0 为 72 名受试者随机产生随机数和随机号，完成随机区组），分别为治疗组和对照组。

2.3 盲法

由于针刺操作的特殊性，不对操作者施盲。疗效评价采用盲法评价，由不知分组信息的人员完成；资料总结阶段采用盲法统计分析，由不知分组情况的统计

人员对研究结果进行统计。从而实行研究操作者、疗效评估者、结果统计者三者分离。

2.4 治疗方法

（1）继续服用针刺干预前的药物。

（2）选穴

治疗组穴位处方：百会、印堂、神门、膻中、内关、太冲

对照组穴位处方：非经非穴；背部，第三胸椎至第七胸椎棘突下，后正中线旁开 2 寸

（3）针具选择

华佗牌一次性针灸针 φ0.25×40mm，针具购自苏州医疗用品厂有限公司（生产企业许可证：苏食药监械生产许 2001-0020 号；注册证号：苏食药监械（准）字 2004 第 2270202 号）。

（4）操作方法

① 治疗组操作：患者仰卧位，局部皮肤常规消毒后，选用毫针针刺，百会、印堂、膻中平刺 0.3-0.8 寸，神门、内关、太冲直刺 0.3-1 寸，得气后留针 30min。治疗频次及疗程：每日治疗 1 次，每周治疗 2 次，4 次为一个疗程。治疗 2 个疗程。

② 对照组操作：患者俯卧位，局部皮肤常规消毒后，选用 Streitberger 针具对背部双侧共 8 个穴位进行“针刺”。治疗时间、疗程同治疗组。

3 观察指标

（1）主要结局指标：汉密尔顿焦虑量表(HAMA)

（2）次要结局指标：匹兹堡睡眠质量指数（PSQI）及焦虑自评量表（SAS）

HAMA 量表包括 14 个项目，所有项目采用 0-4 分的 5 级评分法。HAMA 总分能较好的反应焦虑症状的严重程度，PSQI 用于评估患者睡眠质量，PSQI 主要有睡眠质量、睡眠时间、睡眠障碍等 7 个维度，共 18 个积分自评条目构成，每个维度均采用 0-3 分的 4 级评分法。SAS 用于评估患者的不良症状。采用尼莫地平法[（治疗前总积分-治疗后总积分）/治疗前总积分]*100%，计算减分率。

临床缓解：焦虑情绪及伴随症状消失，减分率≥75%。

显效：焦虑情绪或伴随症状明显减轻，减分率≥50%，＜75%。

好转：焦虑情绪或伴随症状有所减轻，减分率≥25%，＜50%。

无效：焦虑情绪或伴随症状无明显变化，甚至加重，减分率＜25%。

（3）研究周期与评价时间点

本课题治疗期为 4 周，随访期为 2 个月。

HAMA、PSQI 及 SAS 的评价时间包括以下 4 个节点：①基线测定；②治疗期评价 II（第二个疗程结束）；③随访期评价（随访一个月末、两个月末）。

（4）安全性指标

① 不良事件的记录

对不良事件的出现，主要通过受试者随时自觉地反馈，对此事先要指导受试者。出现不良事件时，应将不良事件出现日期及消失日期，不良事件的程度与结果，对所进行的疗法采取的措施及与所进行的疗法因果关系，除疗法之外可能诱发不良事件的可疑药物或处置，对不良事件采取治疗措施与否及其具体内容等，均要作详细记录。

② 不良事件的处理

针刺常见不良事件如晕针、滞针、弯针、断针、针后异常感、出血和皮下血肿、针刺腧穴处疼痛及其他异常情况的处理，依据中国针灸学会国家行业标准的要求，采取适当的措施。

（5）依从性评价

患者依从性采用治疗次数计数法，以评估受试者的依从性。计算公式如下：

治疗依从性=受试者已接受治疗次数/受试者应接受治疗总次数*100%

4 统计分析

采用 SPSS 25.0 软件分析，所有的统计检验均采取双侧检验，$P<0.05$ 时认为有统计学意义，$P<0.01$ 时认为有显著统计学意义。在数据描述方面，计量资料采用均数±标准差（$\bar{x} \pm s$）表示，计数资料和等级资料采用构成比（%）表示。计量资料比较，若符合正态性和方差齐性，两两比较采用独立样本 T 检验，自身前后比较采用配对 T 检验；若不符合正态性和方差齐性，两两比较采用 Mann-Whitney U 秩和检验，自身前后比较采用 Wilcoxon 配对秩和检验。

5 技术路线

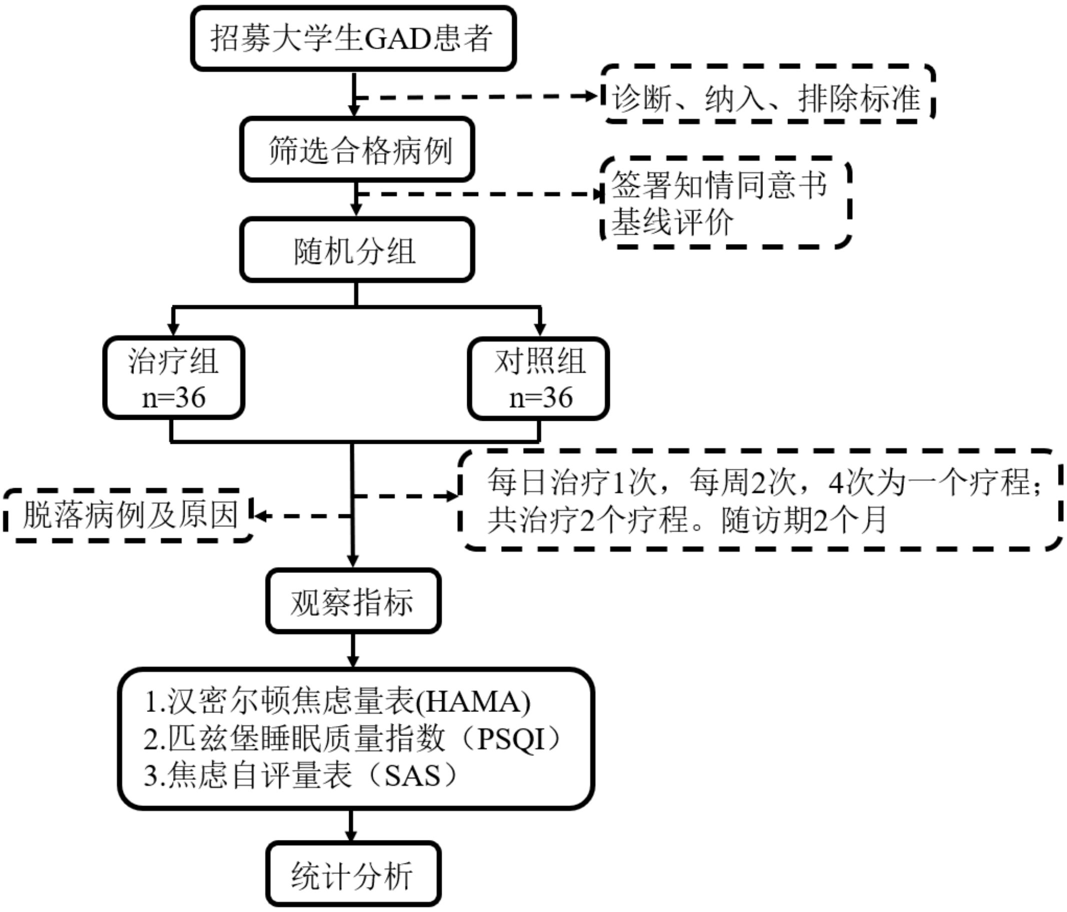
招募大学生GAD患者
诊断、纳入、排除标准
筛选合格病例
签署知情同意书
基线评价
随机分组
治疗组
n=36
对照组
n=36
每日治疗1次，每周2次，4次为一个疗程；
共治疗2个疗程。随访期2个月
脱落病例及原因
观察指标
1.汉密尔顿焦虑量表(HAMA)
2.匹兹堡睡眠质量指数（PSQI）
3.焦虑自评量表（SAS）
统计分析
