# Supplementary material for: The efficacy of acupuncture for generalized anxiety disorder (GAD) in college students: Study protocol for a randomized controlled trial
Source: PLoS One. 2025 Jan 9;20(1):e0316804. doi: 10.1371/journal.pone.0316804 (PMC11717188; doi:10.1371/journal.pone.0316804)

Research Protocol

A randomized controlled study of the effect of acupuncture on the efficacy of treatment of generalized anxiety disorder in college students

Department of Acupuncture and Moxibustion, the Third Affiliated Hospital of Zhejiang Chinese Medical University

Research prospect analysis

Anxiety can be an appropriate response to stress, but when it is difficult to control, it is considered a pathological disease. Generalized Anxiety Disorder (GAD) is the most common type of anxiety disorder, characterized by persistent tension and may be accompanied by hyperactivity of the autonomic nervous system and increased alertness in chronic psychiatric disorders. This disease is a common disabling disease that is often underestimated and not adequately treated; [1] This can lead to heavy personal pain and economic burden.

GAD usually occurs during adolescence or early adulthood, and the incidence of GAD among young people enrolled in university is higher than that of ordinary adults [3]. The World Health Organization's International College Student Project on Mental Health surveyed approximately 14000 college students from 8 countries and found that the prevalence of GAD is about 18%, second only to severe depression. The survey results of 621 university psychological counseling centers in 2018 showed that anxiety is the main reason for students to visit campus psychological health counseling centers. The high prevalence of GAD among college students may reflect the transition from adolescence to adulthood, characterized by entering a more independent living environment, increasing personal decision-making responsibilities, and having non family living partners; [5] Anxiety during college is related to smoking, sleep problems, and even poor academic performance. Moreover, the occurrence of anxiety disorder in early adulthood increases the risk of developing other neurological disorders in the future [7]. Therefore, preventing and treating GAD among college students has significant public health implications [8].

The occurrence of GAD is mainly caused by a combination of genetic and environmental effects, and is related to socio-cultural, individual physical and psychological factors [9, 10]. Modern medicine believes that its pathogenesis is related to certain neurotransmitters, such as 5-hydroxytryptamine and γ-aminobutyric acid; [11] abnormal activation of prefrontal and temporal lobe functions in the brain; [12, 13] and neuroendocrine dysfunction [14].

The definition of depression in traditional medicine is a class of illnesses characterized by depression, restlessness, fullness in the chest, fullness in the ribs, or easy to cry, or choking in the pharynx, such as a foreign body. The etiology of the disease is always emotional and emotional injuries, the onset of the closest relationship with the liver, involving the heart, spleen, kidney; the basic pathogenesis is stagnant qi, visceral yin and yang, qi and blood imbalance [15]. Traditional medicine does not have the term "generalized anxiety disorder", but the ancient literature recorded in the "plum nucleus gas", "dirty mania", "lily disease" and so on belong to this syndrome. "and so on all belong to the category of this evidence. As early as in the Yellow Emperor's Classic of Internal Medicine, there is a record of the disease caused by emotions and feelings, "People have five collections of five qi, in order to generate joy, anger, sadness, worry and fear. Therefore, joy and anger hurt qi, cold and heat hurt form. Violent anger injures yin, violent joy injures yang." "All diseases are born in the qi, anger is qi up, joy is qi slowing down, sorrow is qi eliminating, fear is qi down, cold is qi collecting, gui is qi leaking, shock is qi disruption, labor is qi depletion, and thought is qi knotting."

Plum nucleus qi was first described in "Nanyang Living People's Book" and "Golden Chamber Essentials" as "like having a sizzling sliced meat in the throat". Jin Gui Yao Liao records that "the woman's dirty agitation, like sadness and desire to cry, like the gods, a number of under stretching, sweet wheat and jujube soup Lord"; "Lily disease, the hundred veins of a clan, all cause their disease. I want to eat, but cannot eat ...... if there is a god, the body shape as and, the pulse is slightly counted".

At present, the main treatment methods used for this disease include pharmacological treatment and non-pharmacological treatment; [10] pharmacological therapy has many shortcomings while achieving certain therapeutic efficacy, more adverse reactions, long-term oral easy to lead to increased drug resistance and addictive, and at the same time easy to stop the drug withdrawal reaction, resulting in poor adherence to the therapy; non-pharmacological therapy includes psychotherapy, cognitive behavioral therapy, relaxation therapy, etc., non-pharmacological treatment is affected by many factors and the efficacy is unstable, and it is difficult for patients to adhere to treatment for a long period of time [16]. Non-pharmacological therapies include psychotherapy, cognitive behavioral therapy, relaxation therapy, etc. Non-pharmacological therapies are affected by many factors and have unstable efficacy, making it difficult for patients to adhere to the treatment for a long time [16]. Therefore, it is the research trend to seek a good compliance, safe and effective treatment method.

Acupuncture and moxibustion is one of the current treatments for GAD [17, 18]. As early as in Huangfu Mi's acupuncture and moxibustion Classics A and B in the Western Jin Dynasty, there were 85 acupoints for the treatment of emotional diseases. For example, the head of Neiguan acupoint was "afraid but good at panic", and Rangu acupoint was "afraid of being caught". Tang Sun Simiao proposed the "Thirteen Needles of Ghost Acupoints" for Qu Ze, the head of Da Ling, who had a calm and joyful heart, and the head of Shaofu, who had a fear of palpitations and insufficient qi; Moreover, the Thirteen Ghost Points are still highly valued in the treatment of various mental illnesses to this day. In recent years, clinical trials have also shown that acupuncture and moxibustion has a certain effect on GAD. Yao Xiaoyan et al. [19] analyzed the acupoint selection rules for acupuncture treatment of GAD and concluded that ① emphasizing the treatment of the heart and brain, ② selecting the main acupoints as Baihui, Shenmen, Neiguan, Sanyinjiao, and Yintang, ③ selecting acupoints along the meridian mainly includes the Du meridian, Foot Taiyang Bladder Meridian, Hand Jueyin Pericardium Meridian, and Hand Shaoyin Heart Meridian. But there are also studies [20] indicating that acupuncture has no therapeutic effect on GAD.

In conclusion, the present study selected patients with important health economics who are current college students as the research subjects, with a view to evaluating the efficacy of acupuncture in the treatment of GAD in college students and its stability.

References

[1] DeMartini J, Patel G, and Fancher TL. Generalized Anxiety Disorder[J]. Ann Intern Med, 2019, 170(7):49-64.

[2] Baldwin D, Woods R, Lawson R, et al. Efficacy of drug treatments for generalised anxiety disorder: systematic review and meta-analysis[J]. BMJ, 2011, 342:1199.

[3] Lijster JM, Dierckx B, Utens EM, et al. The Age of Onset of Anxiety Disorders[J]. Can J Psychiatry, 2017, 62(4):237-246.

[4] Auerbach RP, Mortier P, and Bruffaerts R, et al. WHO World Mental Health Surveys International College Student Project: Prevalence and distribution of mental disorders[J]. J Abnorm Psychol, 2018, 127(7):623-638.

[5] Byrd-Bredbenner B, Eck K, and Quick V. Psychometric Properties of the Generalized Anxiety Disorder-7 and Generalized Anxiety Disorder-Mini in United States University Students[J]. Front Psychol, 2020, 24(11):550533.

[6] Bartolo A, Monteiro S, and Pereira A. Factor structure and construct validity of the Generalized Anxiety Disorder 7-item (GAD-7) among Portuguese college students[J]. Cad Saude Publica, 2017, 33(9):e00212716.

[7] Strohle A, Gensichen J, and Domschke K. The Diagnosis and Treatment of Anxiety Disorders[J]. Dtsch Arztebl Int, 2018, 155(37):611-620.

[8] Kanuri N, Taylor CB, Cohen JM, et al. Classification models for subthreshold generalized anxiety disorder in a college population: Implications for prevention[J]. J Anxiety Disord, 2015, 34:43-52.

[9] Xinbao. Neuropsychiatry [M]. Beijing：Science and Technology Press,1980: 87.

[10] Hoge EA, Ivkovic A, and Fricchione GL. Generalized anxiety disorder: diagnosis and treatment[J]. BMJ, 2012, 345: e7500.

[11] Marcinkiewcz CA, Mazzone CM, D'Agostino G, et al. Serotonin engages an anxiety and fear-promoting circuit in the extended amygdala[J]. – Nature, 2016, 537(7618):97-101.

[12] Ball TM, Ramsawh HJ, Campblee-Sills L, et al. Prefrontal dysfunction during emotion regulation in generalized anxiety and panic[J]. Psychol Med, 2013, 43(7):1475-86.

[13] Robinson OJ, Krimsky M, Lieberman L, et al. Towards a mechanistic understanding of pathological anxiety: the dorsal medial[J]. Lancet Psychiatry, 2014, 1(4):294-302.

[14] Hibert K, Lueken U, and Beesdo-Baum K.H. Neural structures, functioning and connectivity in Generalized Anxiety Disorder and interaction with nerurendocrine systems: a systematic review[J]. J Affect Disord, 2014, 158:114-26.

[15] Mianhua Wu, Xinyue Wang. Traditional Chinese Medicine Internal Medicine [M]. 3rd edition. Beijing: China Traditional Chinese Medicine Press, 2014:351-358.

[16] Jing Zhao, Yaping Quan, Yuantao Chen. Progress in Traditional Chinese Medicine Research on Generalized Anxiety Disorder [J]. Journal of Liaoning University of Traditional Chinese Medicine, 2015, 17(04): 210-212.

[17] Xueping Yu, Gengxin Zhang. Observation on the therapeutic effect of Shuigou acupoint on generalized anxiety disorder [J] Shanghai acupuncture and moxibustion Journal [J]. Shanghai Journal of acupuncture and moxibustion and Moxibustion, 2016, 35(02):162-164.

[18] Yan Zhao, Wei Zou, Wei Teng, et al. Clinical Study on the Treatment of Generalized Anxiety Disorder with Tongdu Tiaoshen Acupuncture Method [J]. Clinical Journal of acupuncture and moxibustion and Moxibustion, 2014, 30(11): 24-26.

[19] Xiaoyan Yao, Jun Liu, Shanshan Li, et al. A Study on Acupuncture Treatment of Generalized Anxiety Disorder and Point Selection Rules [J]. Chinese Journal of Traditional Chinese Medicine Information, 2017, 24(07): 94-97.

[20] Mark AD, Chung VCH, Yuen SY, et al. Noneffectiveness of electroacupuncture for comorbid generalized anxiety disorder and irritable bowel syndrome[J]. J Gastroenterol Hepatol, 2019,

34(10):1736-1742.

Research content

Mainly describe the research content, proposed solutions to key problems, expected goals, and main innovative points of the project.

1 Research content

This study focuses on college students with GAD and intends to collect patients who meet the inclusion criteria. The patients will be randomly divided into two groups for treatment, namely the acupuncture group and the control group, using a randomized controlled trial (RCT) method. The efficacy of acupuncture treatment for GAD will be evaluated using the Hamilton Anxiety Scale (HAMA), Pittsburgh Sleep Quality Index (PSQI), and Self Rating Anxiety Scale (SAS) to explore the efficacy of acupuncture treatment.

2 Key questions to be addressed

Whether acupuncture is an effective method in the treatment of GAD in college students.

3 Expected Objectives

To clarify whether acupuncture can be an effective method in treating GAD in college students and the stability of its efficacy.

4 Main Innovations

This study observes the changes of various symptom scales of college students with GAD before and after acupuncture treatment, and provides a certain basis for whether acupuncture can improve the symptoms of college students with GAD.

Research methods and technical roadmap

Mainly describe the research methods, technical roadmap, and feasibility analysis of the project.

1 Research Methods

1.1 Research subjects

1.1.1 Source of subjects

The subjects of this study are all patients from the acupuncture and moxibustion Department of our hospital who meet the criteria of intention.

1.1.2 Diagnostic criteria

(1) Western medical diagnostic criteria refer to Diagnostic and Statistical Manual of Mental Disorders, Fifth Edition (DSM-5) [S]. Beijing: Peking University Press, 2015.7, the diagnostic criteria of GAD:

① Individuals find it difficult to control this concern;

② This anxiety and worry are related to at least three of the following six symptoms: 1. Sitting restless or feeling excited or nervous. 2. Easy to get tired. 3. Difficulty concentrating or blank mind. 4. Easy to provoke. 5. Muscle tension. 6. Sleep disorders.

③ This anxiety, worry, or physical symptom causes clinically significant pain, or leads to damage in social, occupational, or other important functional aspects.

④ This obstacle cannot be attributed to the physiological effects of certain substances or other physical diseases.

⑤ This disorder cannot be better explained by other mental disorders.

⑥ For most days of at least 6 months, excessive anxiety and concern are exhibited towards various events or activities.
(2) Diagnostic criteria of Chinese medicine refer to the diagnostic criteria of "Internal Medicine of Traditional Chinese Medicine", the planning textbook of Chinese medicine colleges and universities across the country:
① The main symptoms are depression, emotional restlessness, chest and hypochondriac distension and pain, or easy to anger and cry, or foreign body sensation in the pharynx, which cannot be spit out or swallowed;
② A history of emotional trauma, anxiety, sadness, fear, resentment and other emotional internal injuries, and recurrent disorders are usually closely related to emotional factors;
③ No other signs and symptoms;
④ Those who meet the above criteria and continue for more than 6 months.

1.1.3 Inclusion criteria

(1) Those who are consistent with the Chinese and Western medical diagnostic criteria of GAD (depression disease);
(2) Those with stable vital signs, conscious and without suicidal tendency;
(3) Undergraduate and graduate students, 18 ≤ age ≤ 30 years old;
(4) 7 ≤ HAMA total score ≤ 21 points;
(5) Those who voluntarily accept the treatment and observation and sign the informed consent.

1.1.4 Exclusion criteria

(1) Those with severe organic pathology or the presence of conditions unsuitable for current treatments;
(2) Those with depression or other psychiatric disorders secondary to anxiety;
(3) Those with abnormal sanity who cannot cooperate;
(4) Pregnant and lactating women;
(5) Those who have participated in other clinical studies in the last 3 months;
(6) Those who cannot tolerate acupuncture treatment.

1.1.5 Exclusion criteria

(1) Subjects with poor compliance and those who did not cooperate with the trial program to complete the treatment after inclusion;
(2) Those who dislodged cases on their own during observation or withdrew from the study on their own.

1.1.6 Abscission criteria

(1) In the course of treatment, there is blood sickness, needle fainting, broken needles, stagnant needles, etc. requesting to stop the treatment failing to complete the course of treatment;
(2) Loss of visit.

1.1.7 Criteria for case discontinuation

(1) Serious complications or serious adverse reactions occur during treatment and it is inappropriate to continue the trial;
(2) Subjects who voluntarily withdraw from the clinical study in the middle of the study due to other external reasons.

2 Research Program

2.1 Sample size estimation

According to the principle of minimum sample size of clinical trials to select 30 subjects in each group, and according to the less than 20% loss of visit rate, 36 subjects were included in each group, and the total study sample was 72 cases.

2.2 Randomization method

The study was conducted using a completely randomized, controlled, single-blind method for the trial, in which 72 subjects were randomly divided into two groups (each subject was numbered according to the sequence of coming to the clinic with serial numbers 001-072, and then SPSS 25.0 was used to generate random numbers and random numbers for the 72 subjects to complete the randomized zones), the treatment group and the control group, respectively.

2.3 Blinding

Due to the specificity of the acupuncture operation, the operator was not blinded. The efficacy evaluation was blinded and completed by personnel who did not know the information of the subgroups; the data summarization stage was blinded and the statistical analysis was performed by statisticians who did not know the subgroups of the results of the study. Thus, there is a separation of the study operator, the efficacy evaluator, and the statistician of the results.

2.4 Treatment

(1) Continue to take the medication you were taking before the acupuncture intervention.

(2) Selection of acupoints

Acupoint prescription for the treatment group: GV20，GV29，HT7，CV17，PC6，LR3

Control group acupuncture point prescription: non-meridian non-acupuncture points; back, the third thoracic vertebrae to the seventh thoracic vertebrae under the spinous process, the posterior median line next to the opening of 2 inches

(3) Needle selection

Hua Tuo brand disposable acupuncture needles φ0.25×40mm, the needles were purchased from Suzhou Medical Supplies Factory Co., Ltd (Manufacturer's License: Suzhou Food and Drug Administration Measures Production Permit No. 2001-0020; Registration No.: Suzhou Food and Drug Administration Measures (Permit) No. 2004 No. 2270202).

(4) Operation method

① Treatment group operation: patients' supine position, local skin routine disinfection, the use of millimetre needle needling, GV20，GV29，CV17 flat prick 0.3-0.8 inches, HT7，PC6，LR3 straight prick 0.3-1 inches, after the gas stay in the needle for 30min. treatment frequency and course of treatment: 1 time a day, 2 times a week, 4 times as a course of treatment. Treatment of 2 courses.

② control group operation: patients lying down, local skin routine disinfection, the use of Streitberger needle on the back of both sides of a total of 8 points "acupuncture". The treatment time and duration are the same as the treatment group.

3 Observation indicators

(1) Primary outcome indicators: Hamilton Anxiety Scale (HAMA)
(2) Secondary outcome indicators: Pittsburgh Sleep Quality Index (PSQI) and Self-Assessment Scale for Anxiety (SAS)
The HAMA scale consists of 14 items, all of which are rated on a 5-point scale from 0-4. The HAMA total score provides a good response to the severity of anxiety symptoms, and the PSQI is used to assess the patient's quality of sleep. The PSQI consists of 7 dimensions, including sleep quality, sleep duration, and sleep disorders, with a total of 18 integral self-assessment entries, each of which is rated on a 4-point scale from 0-3. The SAS is used to assess patients' adverse symptoms. was used to assess patients' adverse symptoms. Nimodipine method [(total points before treatment - total points after treatment)/total points before treatment]*100% was used to calculate the reduction rate.

Clinical remission: anxiety and accompanying symptoms disappeared, score reduction rate ≥75%.
Significant effect: anxiety or accompanying symptoms significantly reduced, score reduction rate ≥50%, <75%.

Improvement: Anxiety or accompanying symptoms are reduced, score reduction rate ≥25%, <50%.

Ineffective: no significant change or even aggravation of anxiety or accompanying symptoms, score reduction rate <25%.
(3) Research period and evaluation time point

The treatment period of this subject is 4 weeks, and the follow-up period is 2 months.

The evaluation time of HAMA, PSQI and SAS included the following 4 nodes: ① baseline measurement; ② evaluation of the treatment period II (end of the second course of treatment); and ③ evaluation of the follow-up period (end of the first month and end of the second month of follow-up).

(4) Safety indicators
① Recording of adverse events

The emergence of adverse events is mainly through the subject's conscious feedback at any time, for which the subject should be instructed in advance. When an adverse event occurs, the date of occurrence and disappearance of the adverse event, the extent and outcome of the adverse event, the measures taken in response to the therapy performed and the causal relationship with the therapy performed, the suspected drugs or treatments other than the therapy that may have induced the adverse event, and whether therapeutic measures have been taken in response to the adverse event and its specific content should all be recorded in detail.
② Treatment of adverse events

The management of common adverse events of acupuncture such as dizzy needles, stagnant needles, bent needles, broken needles, abnormal sensations after needling, bleeding and subcutaneous hematomas, pain at acupuncture points and other abnormalities is based on the requirements of the national industry standards of the Chinese Acupuncture and Moxibustion Association, and appropriate measures are taken.

(5) Evaluation of adherence

Patient adherence was assessed using the number of treatments counting method in order to evaluate the subject's adherence. The calculation formula is as follows:

Treatment adherence = number of times the subject has received treatment / total number of times the subject should receive treatment * 100%

4 Statistical analyses

SPSS 25.0 software was used to analyze the data, and all statistical tests were taken as two-sided tests, with statistical significance considered at $P<0.05$ and statistically significant at $P<0.01$. For data description, measurement information was expressed as mean ± standard deviation ($\bar{x} \pm s$), and count and rank information were expressed as constitutive ratio (%). For the comparison of measurement data, independent samples t-test was used for two-way comparisons and paired t-test was used for before-after self-comparisons if normality and chi-square were met; if normality and chi-square were not met, Mann-Whitney U rank sum test was used for two-way comparisons and Wilcoxon paired rank sum test was used for before-after self-comparisons.

5 Technical approaches

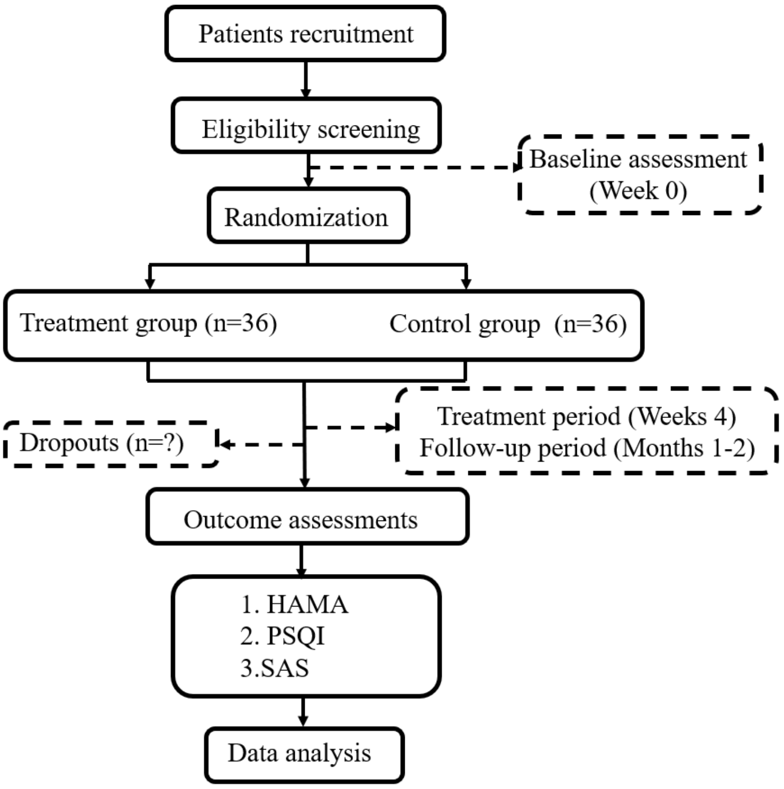

Supplement: S2 File — (PDF) [file pone.0316804.s003.pdf]
